# Supplementary material for: Origin and evolutionary trajectories of brown algal sex chromosomes
Source: Nat Ecol Evol. 2025 Aug 25;9(11):2127–44. doi: 10.1038/s41559-025-02838-w (PMC12592211; doi:10.1038/s41559-025-02838-w)
Supplement: Supplementary file 1 — Supplementary Figs. 1–18. [file 41559_2025_2838_MOESM1_ESM.pdf]

nature ecology & evolution

Supplementary information https://doi.org/10.1038/s41559-025-02838-w

Origin and evolutionary trajectories of brown algal sex chromosomes

In the format provided by the authors and unedited

SUPPLEMENTARY FIGURES

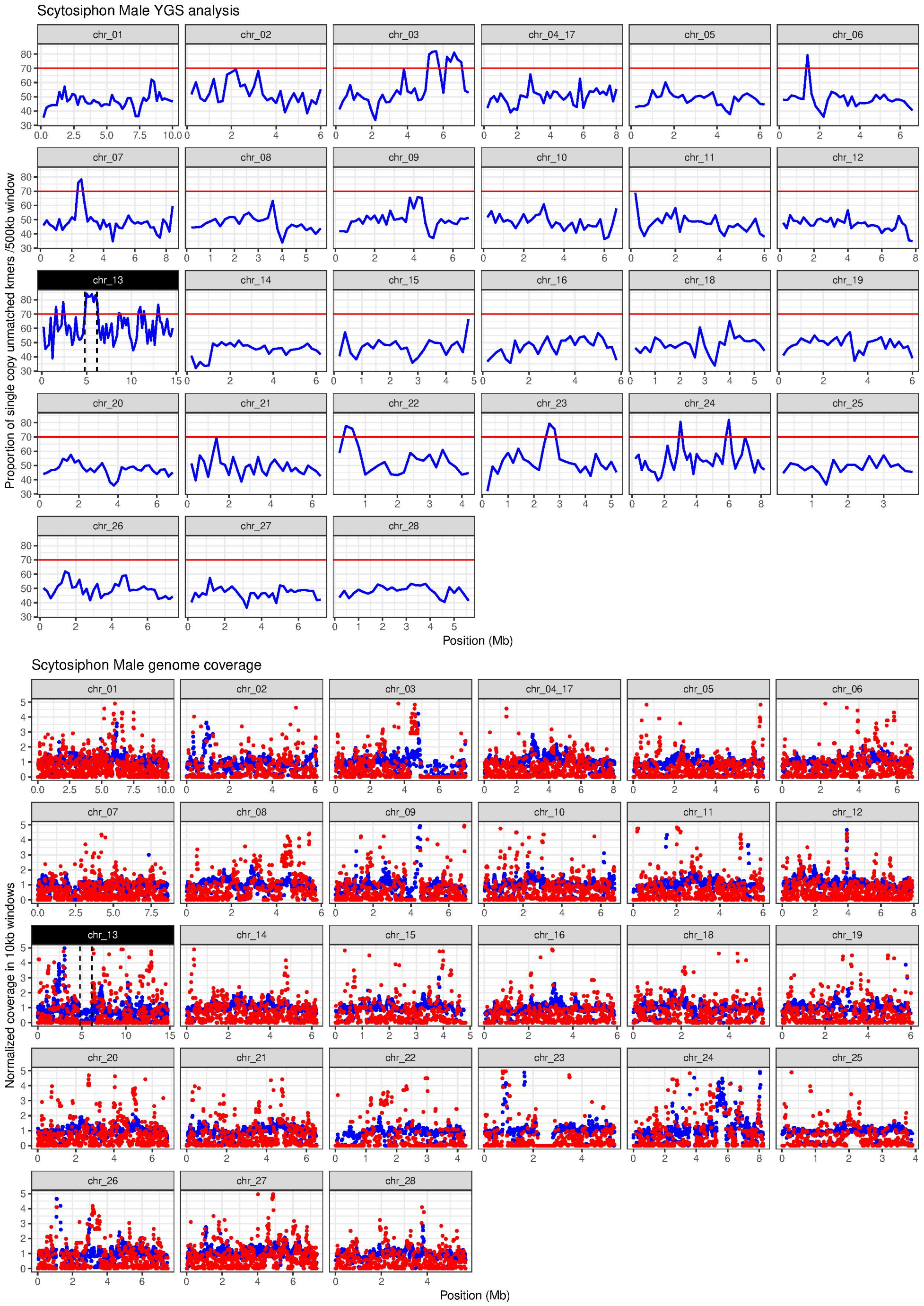

Supplementary Figure 1. *Detection of the sex-determining region (SDR) in the V chromosome of Scytosiphon promiscuus. Results from the k-mer–based YGS analysis (top panels) and genomic read coverage normalized by the genome-wide mean (bottom panels) are shown for each chromosome of the S. promiscuus male genome. Chromosome 13 (chr_13, highlighted with a black header), is homologous to the V chromosome of Ectocarpus sp. 7 and was identified as the sex chromosome. The SDR region, delimited by vertical dashed lines (positions 4,795,133 to 6,159,060), displays a high proportion of unmatched k-mers in the YGS analysis and a clear difference in sequencing read coverage between males (blue) and females (red). The horizontal red line in the YGS plots indicates the threshold of 70% unmatched k-mers used to define putative sex-linked regions.*

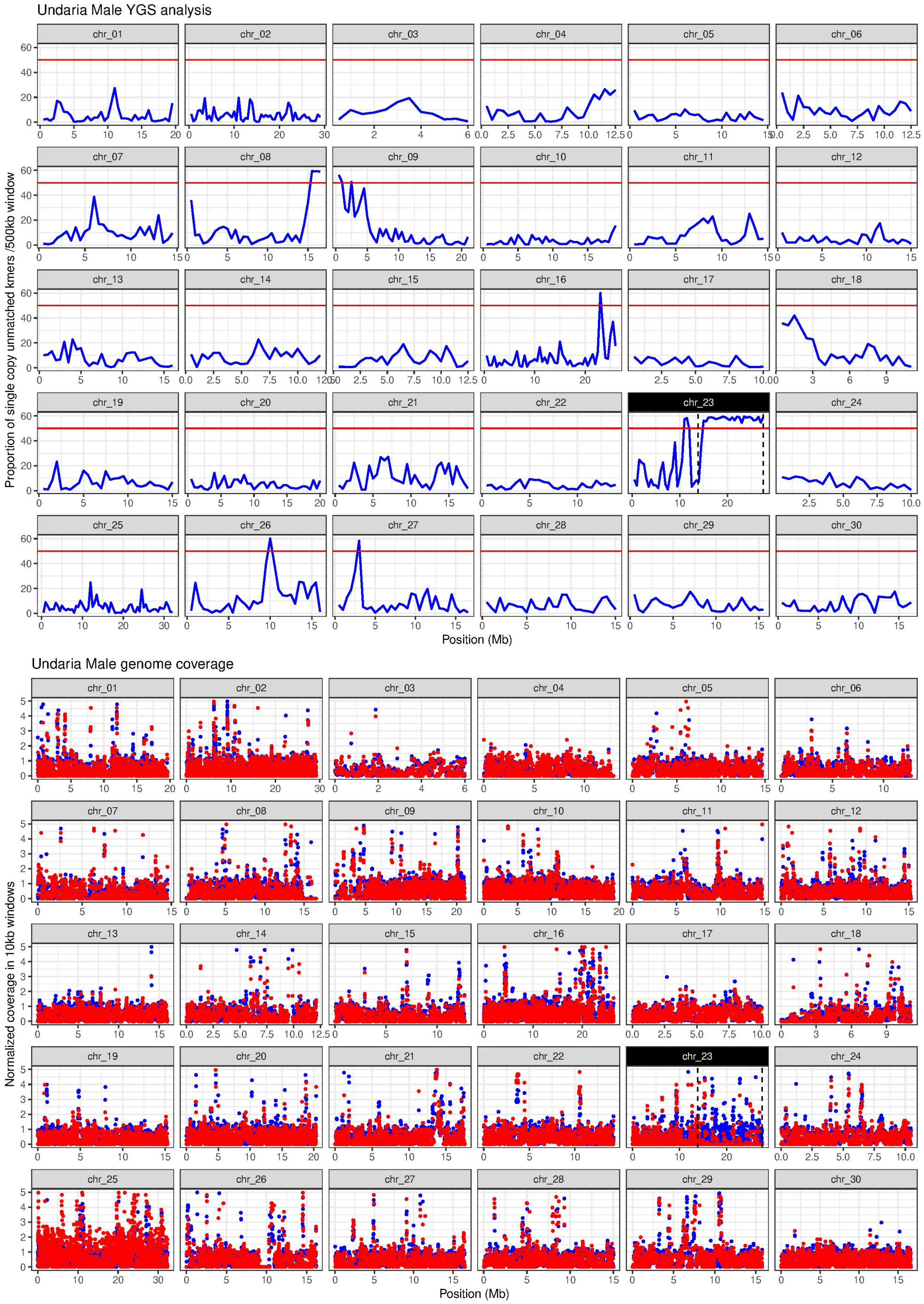

Supplementary Figure 2*. Detection of the sex-determining region (SDR) in the V chromosome of Undaria pinnatifida. Results from the k-mer–based YGS analysis (top panels) and genomic read coverage normalized by the genome-wide mean (bottom panels) are shown for each chromosome of the U. pinnatifida male genome. Chromosome 23 (chr_23, highlighted with a black header), is homologous to the V chromosome of Ectocarpus sp. 7 and was identified as the sex chromosome. The SDR region, delimited by vertical dashed lines (positions 13,867,553 to 27,276,646), displays a high proportion of unmatched k-mers in the YGS analysis and a clear difference in sequencing read coverage between males (blue) and females (red). The horizontal red line in the YGS plots indicates the threshold of 50% unmatched k-mers used to define putative sex-linked regions.*

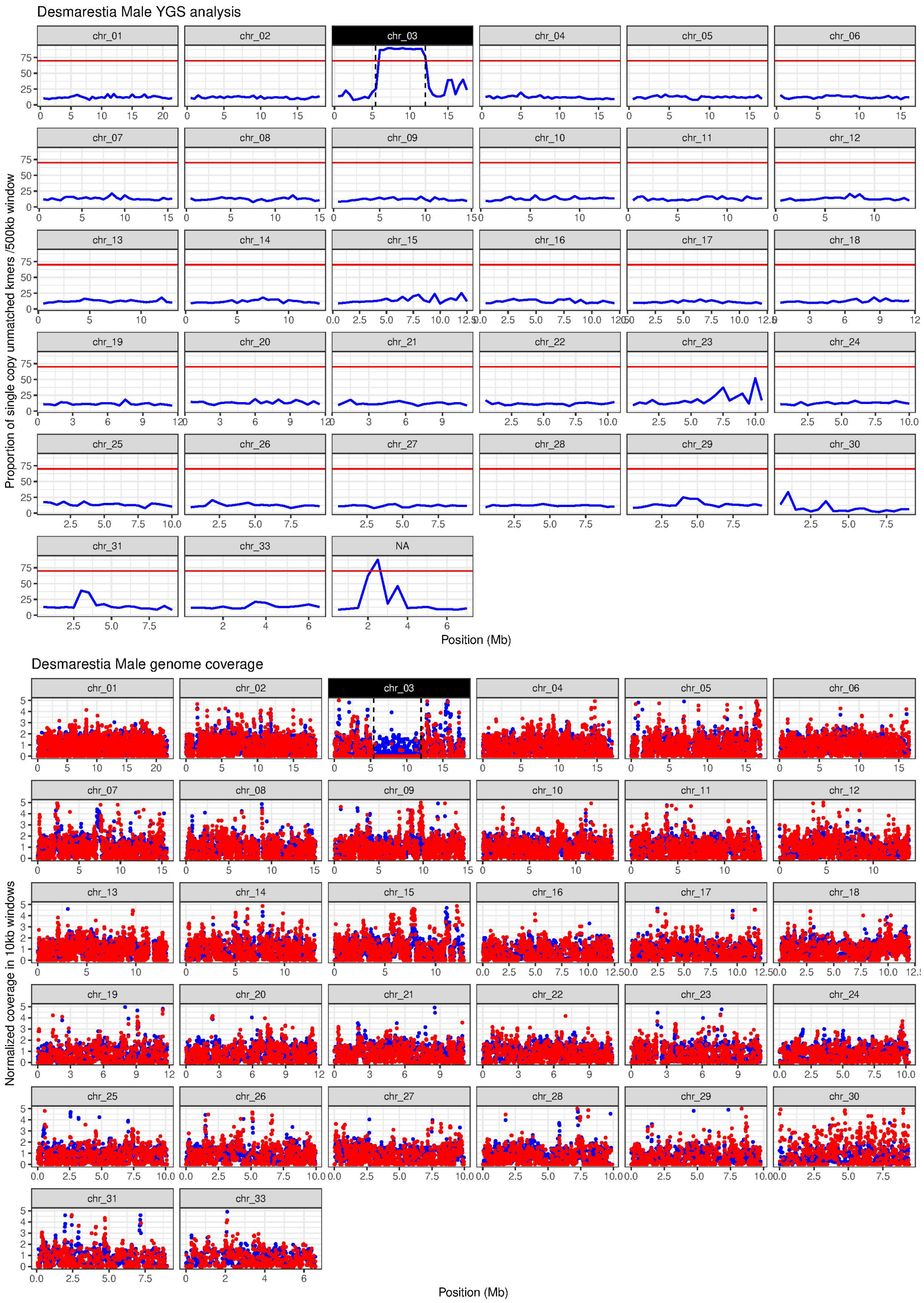

Supplementary Figure 3*. Detection of the sex-determining region (SDR) in the V chromosome of Desmarestia herbacea. Results from the k-mer–based YGS analysis (top panels) and genomic read coverage normalized by the genome-wide mean (bottom panels) are shown for each chromosome of the D. herbacea male genome. Chromosome 3 (chr_03, highlighted with a black header), is homologous to the V chromosome of Ectocarpus sp. 7 and was identified as the sex chromosome. The SDR region, delimited by vertical dashed lines (positions 5,427,118 to 11,995,121), displays a high proportion of unmatched k-mers in the YGS analysis and a clear difference in sequencing read coverage between males (blue) and females (red). The horizontal red line in the YGS plots indicates the threshold of 70% unmatched k-mers used to define putative sex-linked regions.*

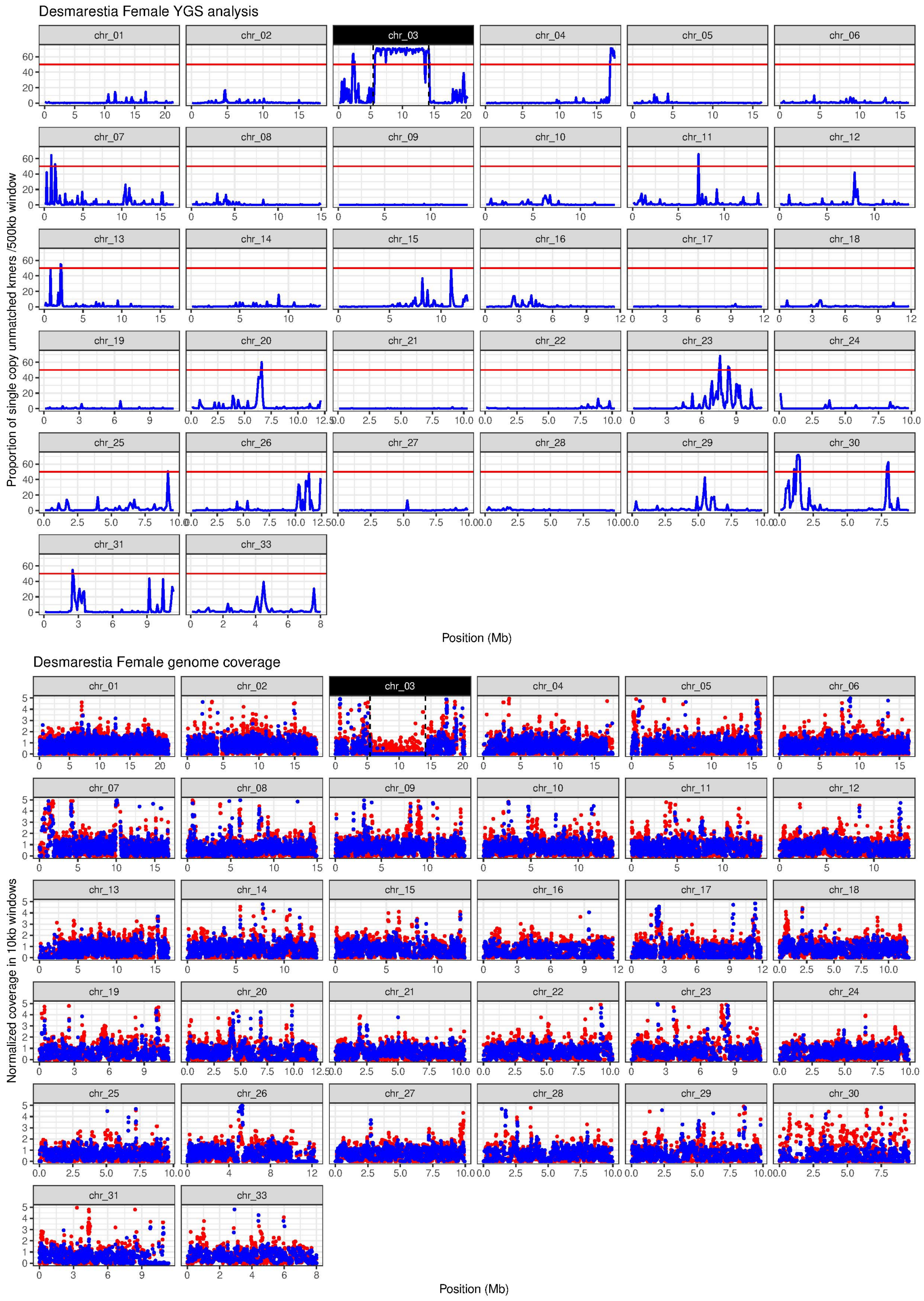

Supplementary Figure 4. *Detection of the sex-determining region (SDR) in the U chromosome of Desmarestia herbacea. Results from the k-mer–based YGS analysis (top panels) and genomic read coverage normalized by the genome-wide mean (bottom panels) are shown for each chromosome of the D. herbacea female genome. Chromosome 3 (chr_03, highlighted with a black header), is homologous to the V chromosome of D. herbacea and was identified as the sex chromosome. The SDR region, delimited by vertical dashed lines (positions 5,479,174 to 14,105,652), displays a high proportion of unmatched k-mers in the YGS analysis and a clear difference in sequencing read coverage between males (blue) and females (red). The horizontal red line in the YGS plots indicates the threshold of 50% unmatched k-mers used to define putative sex-linked regions.*

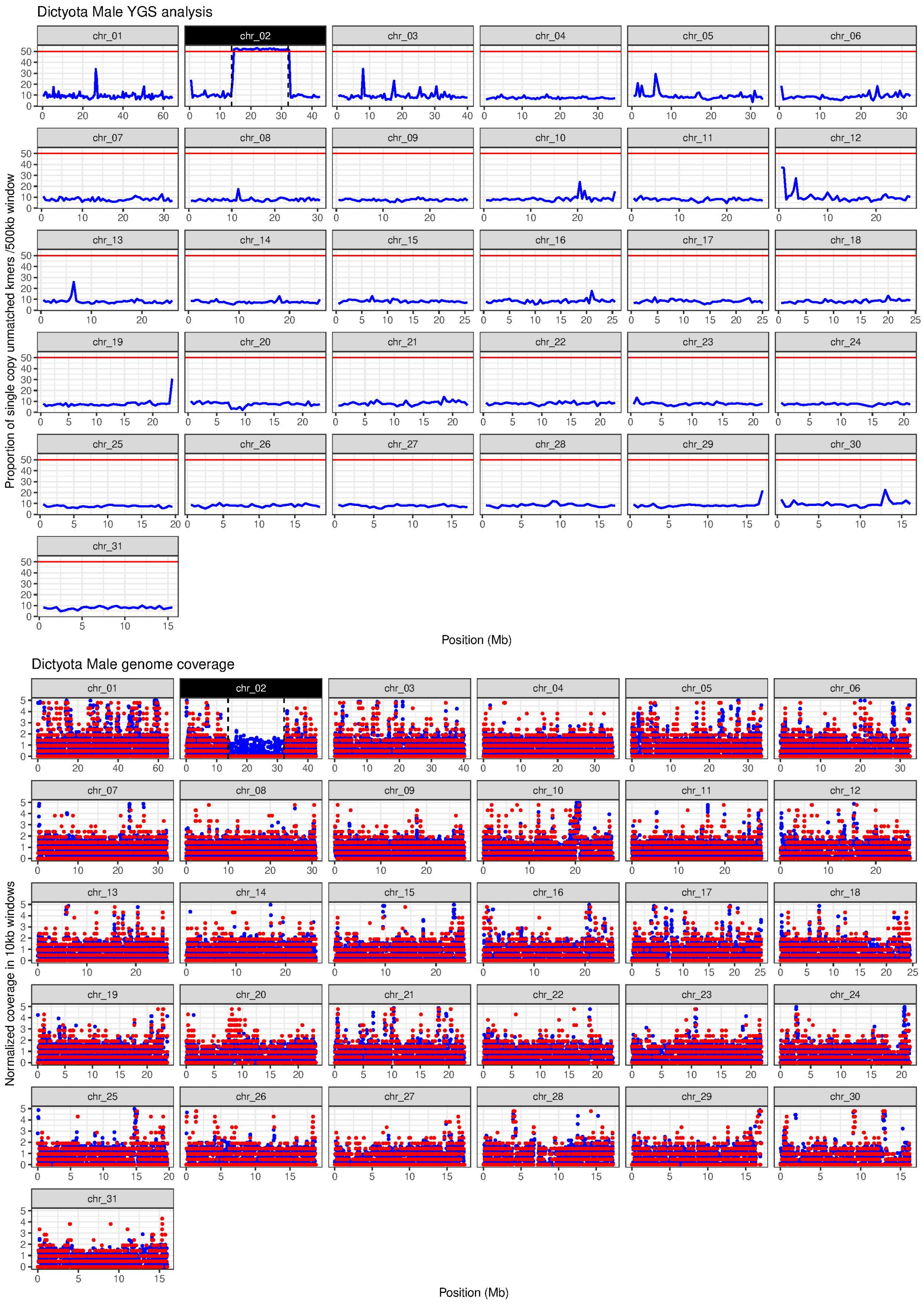

Supplementary Figure 5. *Detection of the sex-determining region (SDR) in the V chromosome of Dictyota dichotoma. Results from the k-mer–based YGS analysis (top panels) and genomic read coverage normalized by the genome-wide mean (bottom panels) are shown for each chromosome of the D. dichotoma male genome. Chromosome 2 (chr_02, highlighted with a black header), is homologous to the V chromosome of Ectocarpus sp. 7 and was identified as the sex chromosome. The SDR region, delimited by vertical dashed lines (positions 14,808,243 to 32,220,768), displays a high proportion of unmatched k-mers in the YGS analysis and a clear difference in sequencing read coverage between males (blue) and females (red). The horizontal red line in the YGS plots indicates the threshold of 70% unmatched k-mers used to define putative sex-linked regions.*

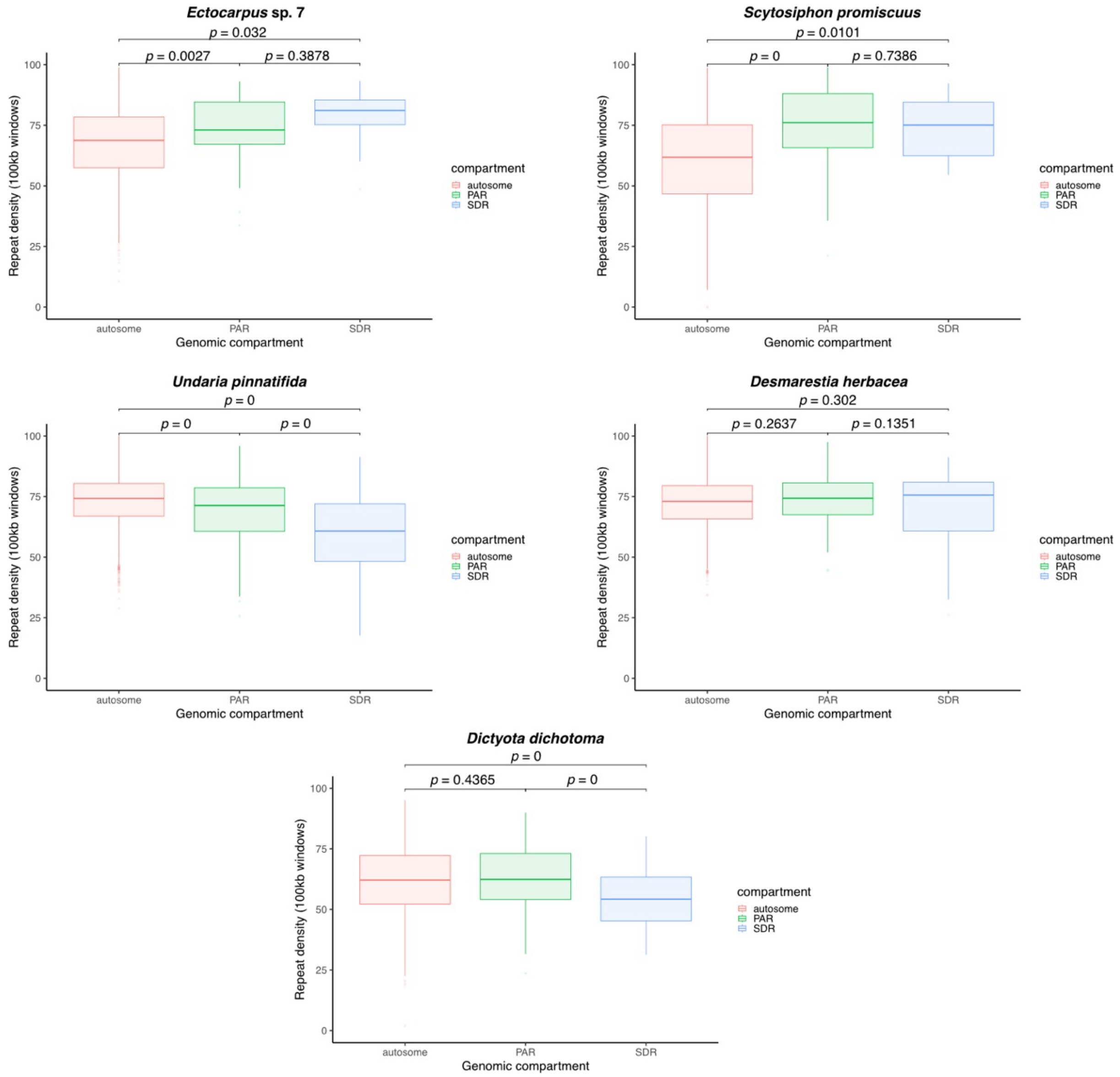

Supplementary Figure 6. *Enrichment in unclassified repeats in the V-SDRs and PARs of Ectocarpus sp. 7 and Scytosiphon promiscuus. Statistically significant differences in mean values of repeat density were assessed using permutation tests.*

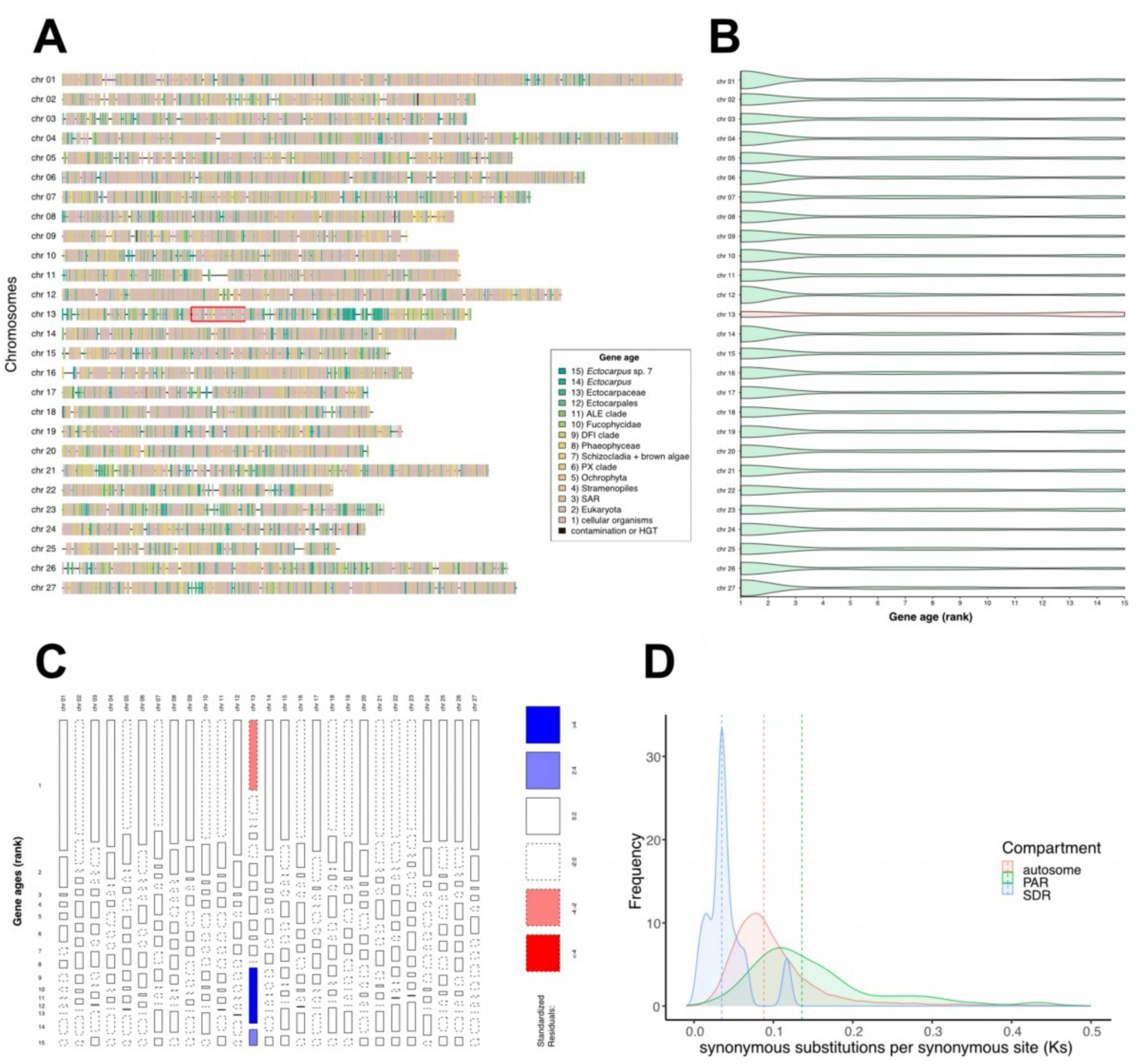

Supplementary Figure 7. *Gene ages across the Ectocarpus sp.7 genome. (A) Distribution of relative gene ages across the chromosomes of Ectocarpus sp. 7. The SDR of the V sex chromosome (chr 13) is highlighted with a red box. (B) The sex chromosome (red) has a significantly higher proportion of young genes and a lower proportion of old genes when compared to the autosomes (green; see Supplementary Table 14). (C) Mosaic plot showing that the species-level (rank 15) and the genus-level (rank 14) genes are responsible for the enrichment of young genes in the sex chromosome. (D) Inter-species Ks values across genomic compartments (autosomes, PARs and SDR) obtained from one-to-one orthologs between Ectocarpus sp. 7 and Ectocarpus siliculosus. The inter-species Ks values are significantly higher in the PARs of the sex chromosome when compared to the autosomes or the SDR (see Supplementary Table 17).*

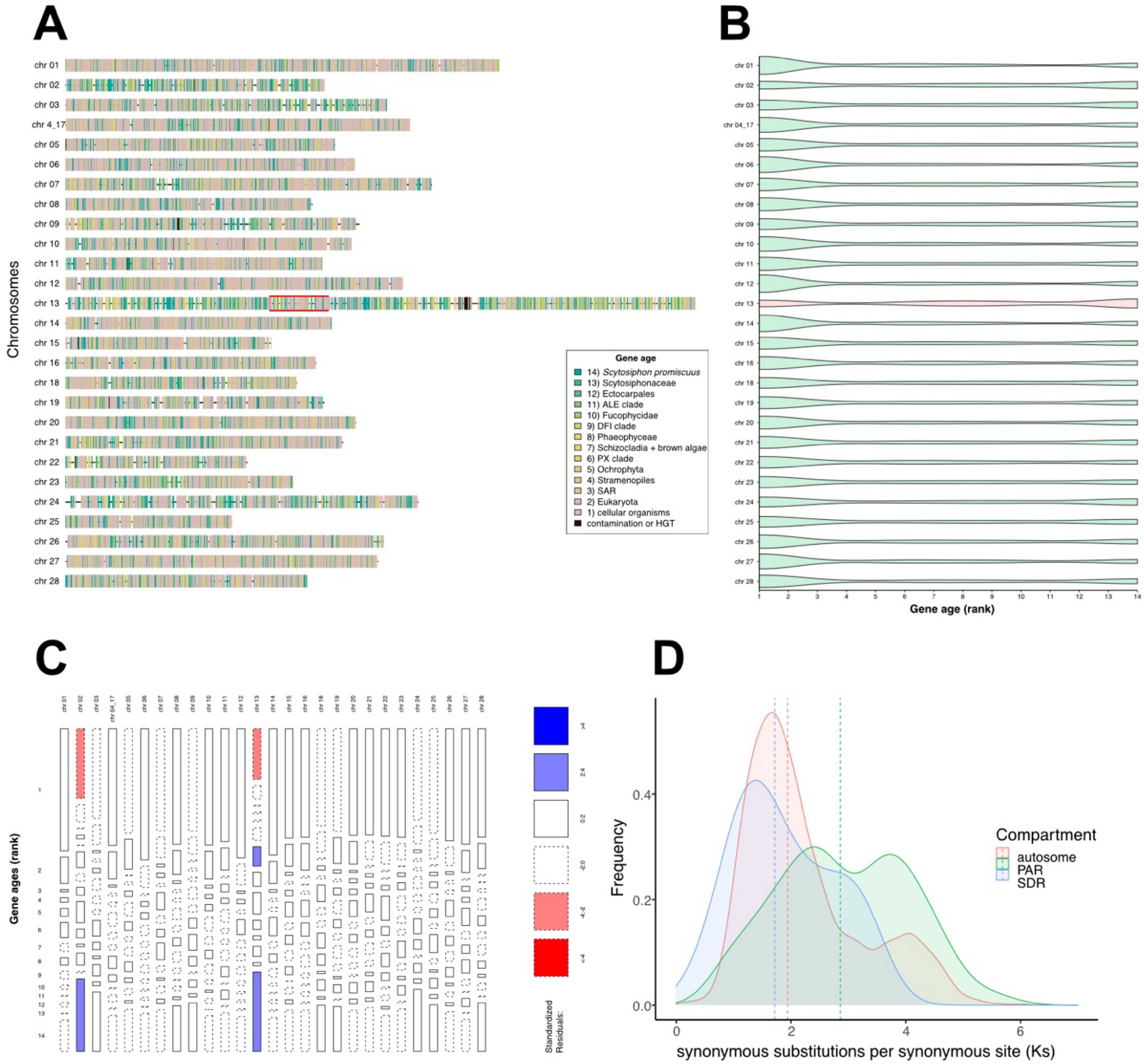

Supplementary Figure 8. *Gene ages across the S. promiscuus genome. A) Distribution of relative gene ages across the chromosomes of Scytosiphon promiscuus. The SDR of the V sex chromosome (chr 13) is highlighted with a red box. (B) The sex chromosome (red) has a significantly higher proportion of young genes and a lower proportion of old genes when compared to most of the autosomes (green; see Supplementary Table 14). (C) Mosaic plot showing that the species-level (rank 14) genes are responsible for the enrichment of young genes in the sex chromosome. (D) Inter-species Ks values across genomic compartments (autosomes, PARs and SDR) obtained from one-to-one orthologs between S. promiscuus and Chordaria linearis. The inter-species Ks values are significantly higher in the PARs of the sex chromosome when compared to the autosomes or the SDR, although most values already reached saturation (see Supplementary Table 17).*

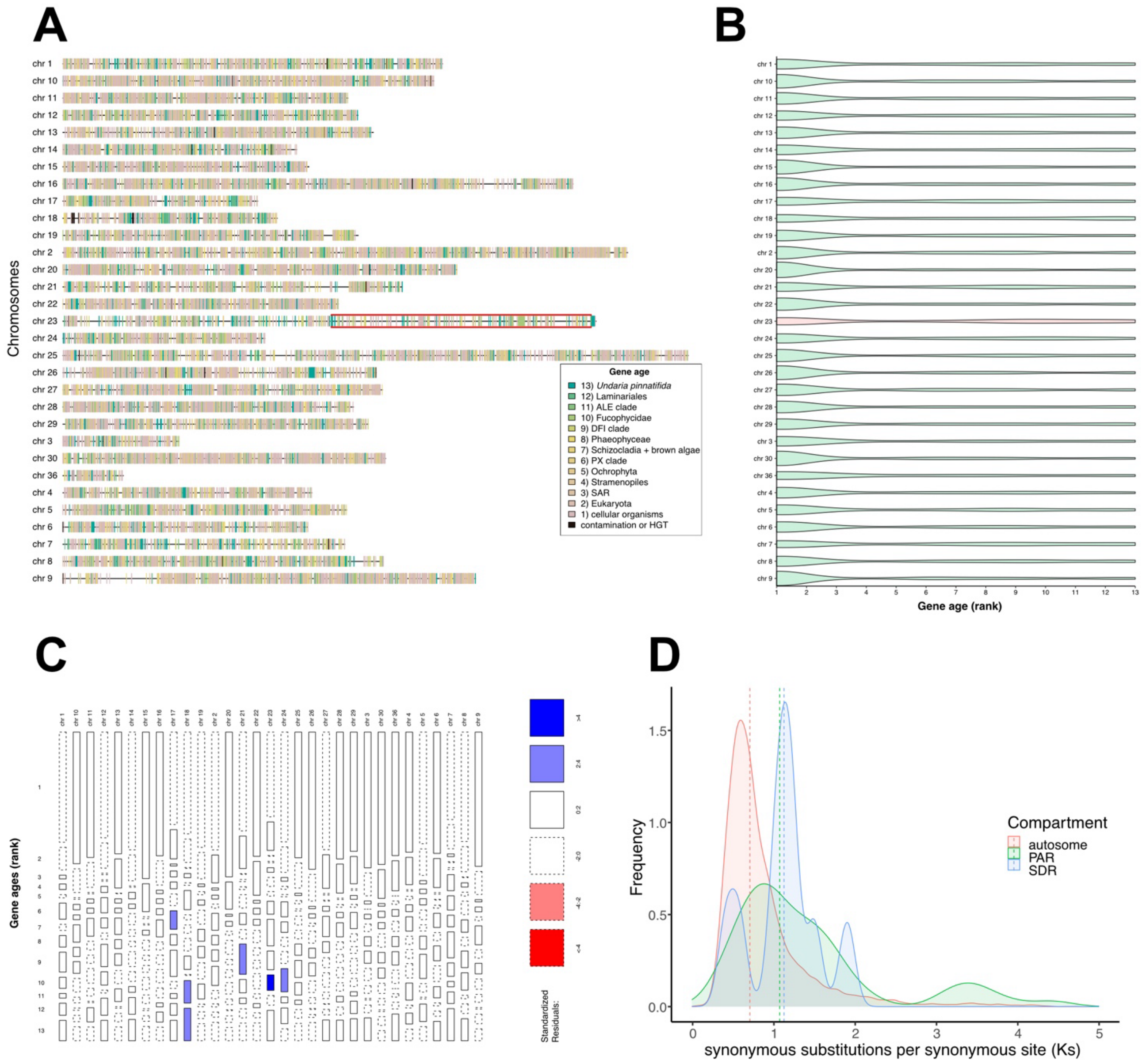

Supplementary Figure 9. *Gene ages across the U. pinnatifida genome. (A) Distribution of relative gene ages across the chromosomes of Undaria pinnatifida. The SDR of the V sex chromosome (chr 23) is highlighted with a red box. (B) The sex chromosome (red) has a significantly higher proportion of young genes and a lower proportion of old genes when compared to most of the autosomes (green; see Supplementary Table 14). (C) Mosaic plot showing that the ALE-clade genes (rank 11) are responsible for the enrichment of young genes in the sex chromosome. (D) Inter-species Ks values across genomic compartments (autosomes, PARs and SDR) obtained from one-to-one orthologs between U. pinnatifida and Saccharina japonica. The inter-species Ks values are significantly higher in the sex chromosome when compared to the autosomes (see Supplementary Table 17), showing similar values in the PARs and in the SDR.*

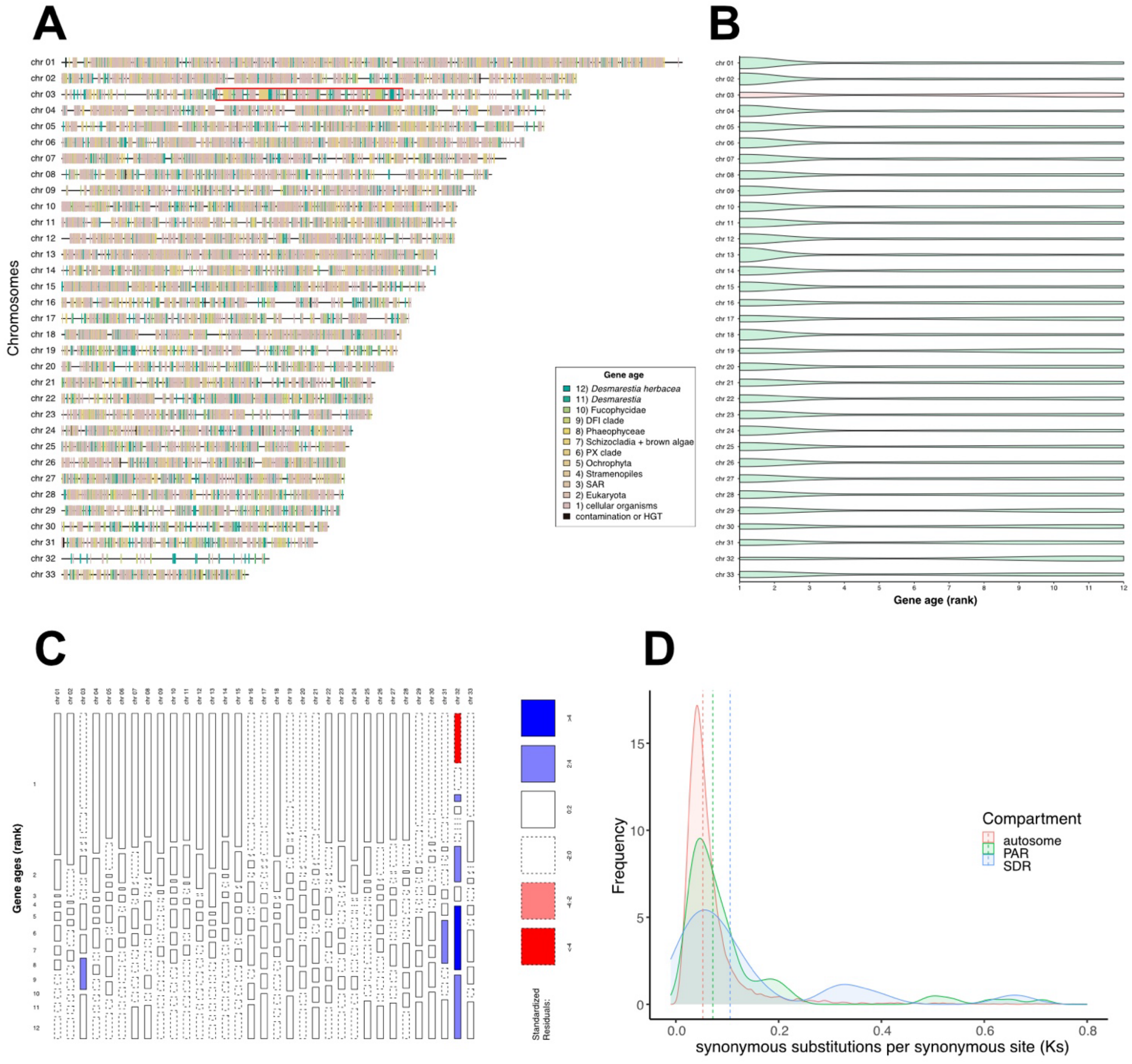

Supplementary Figure 10. *Gene ages across the D. herbacea genome. (A) Distribution of relative gene ages across the chromosomes of Desmarestia herbacea. The SDR of the V sex chromosome (chr 03) is highlighted with a red box. (B) The sex chromosome (red) has a significantly higher proportion of young genes and a lower proportion of old genes when compared to most of the autosomes (green; see Supplementary Table 14). (C) Mosaic plot showing that the genus-level (rank 11) genes are responsible for the enrichment of young genes in the sex chromosome. (D) Inter-species Ks values across genomic compartments (autosomes, PARs and SDR) obtained from one-to-one orthologs between D. herbacea and D. dudresnayi. The inter-species Ks values are significantly higher in the sex chromosome when compared to half of the autosomes (see Supplementary Table 17). Non-significance of inter-species Ks values across chromosomes may be a consequence of the conflation with the Ks values in Desmarestia dudresnayi. The SDR displays higher inter-species Ks values compared to the PARs or the autosomes.*

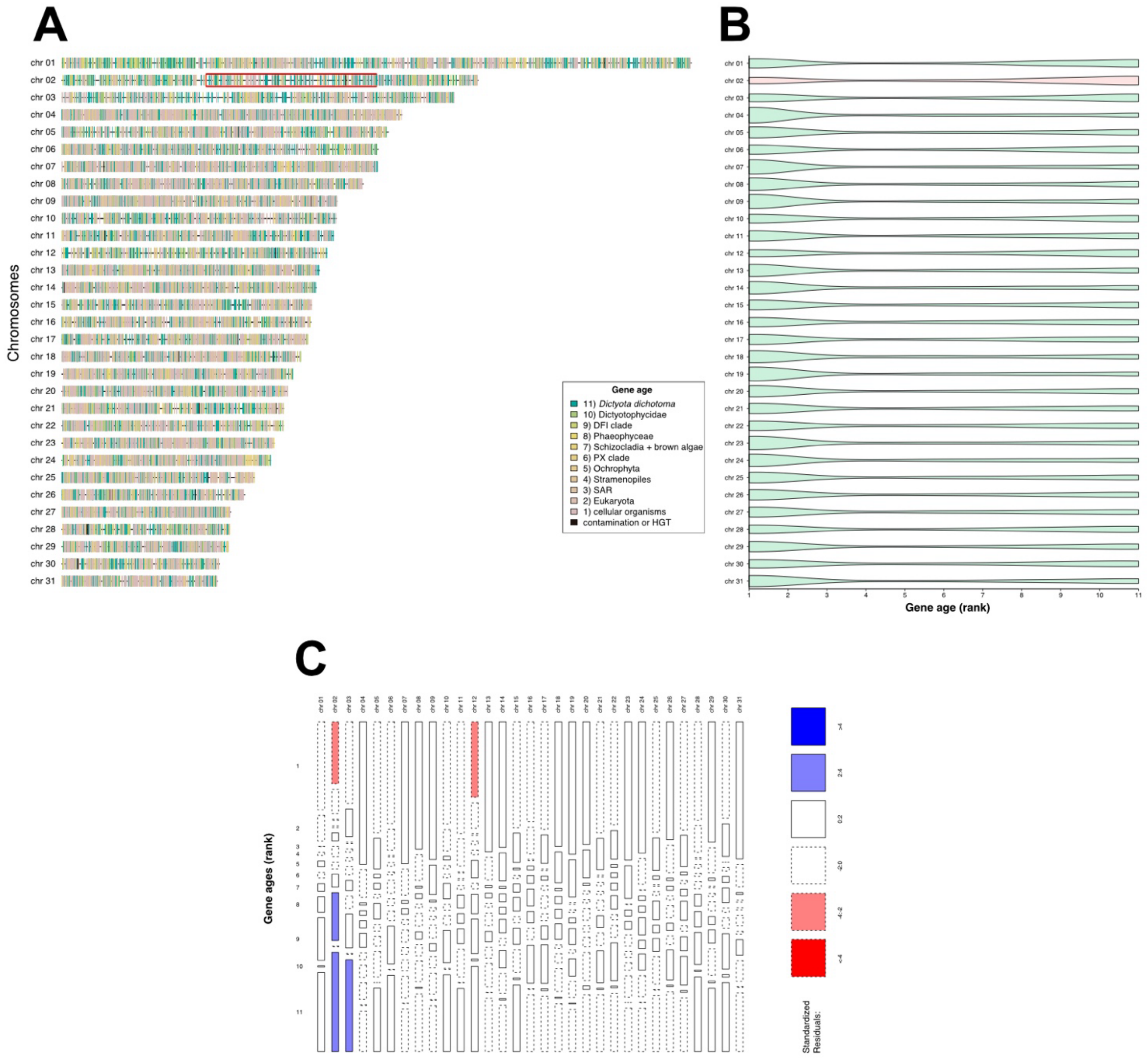

Supplementary Figure 11. *Gene ages across the D. dichotoma genome. (A) Distribution of relative gene ages across the chromosomes of Dictyota dichotoma. The SDR of the V sex chromosome (chr 02) is highlighted with a red box. (B) The sex chromosome (red) has a significantly higher proportion of young genes and a lower proportion of old genes when compared to most of the autosomes (green; see Supplementary Table 14). (C) Mosaic plot showing that the species-level (rank 11) and the DFI-clade-level (rank 9) genes are responsible for the enrichment of young genes in the sex chromosome. Inter-species Ks values were not analyzed for D. dichotoma, due to a saturation of synonymous mutations with the closest species in the PhaeoExplorer database (Halopteris paniculata).*

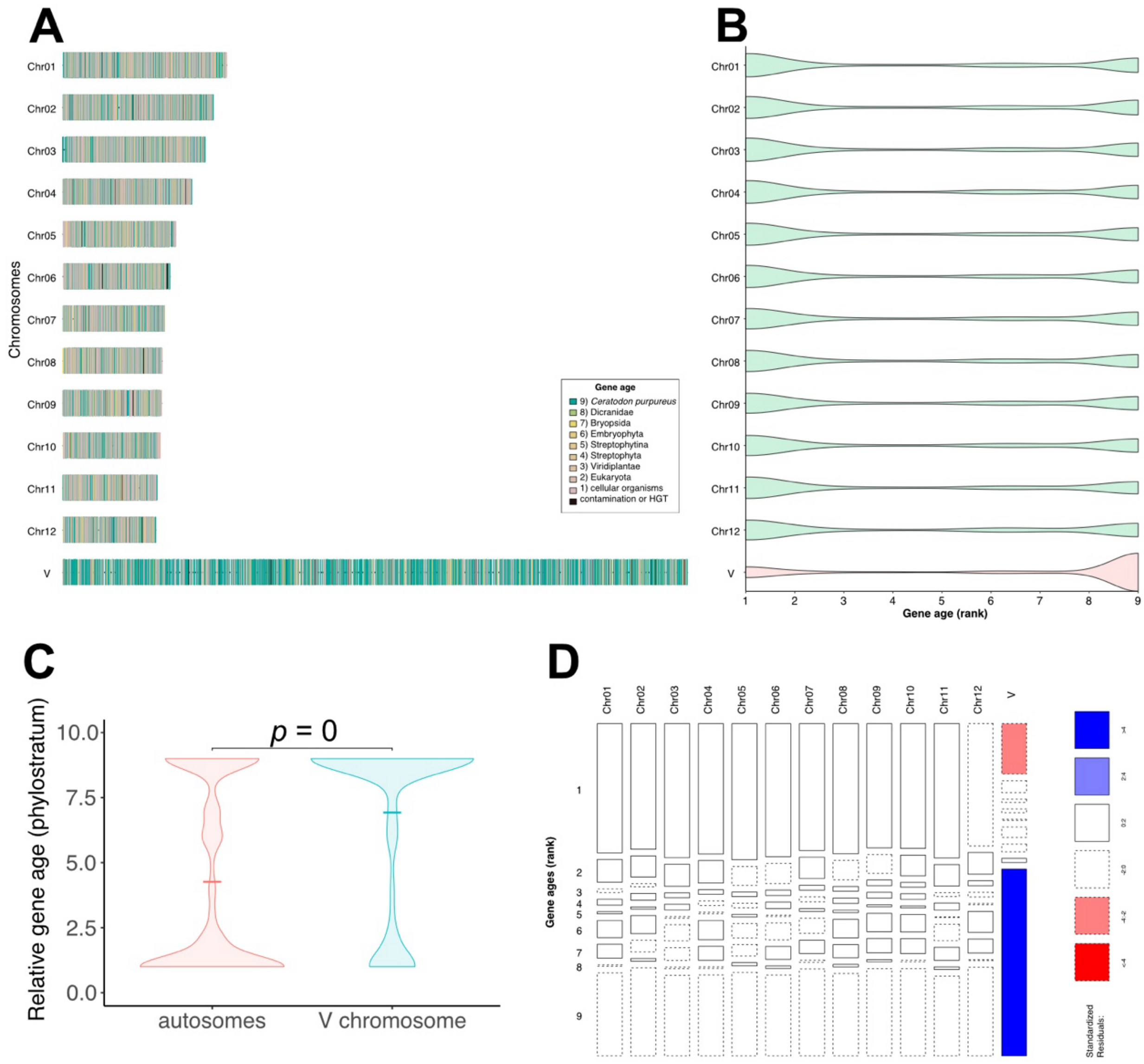

Supplementary Figure 12. *Gene ages across the C. purpureum genome. (A) Distribution of relative gene ages across the chromosomes of C. purpureum. (B) The V sex chromosome (V; red) has a significantly higher proportion of young genes and a lower proportion of old genes when compared to the autosomes (green; see Supplementary Table 14). (C) Violin plot showing the relative gene age ranks (higher ranks equate to younger ages) of the TRGs between the autosomes and the V chromosome**.** Statistically significant difference in mean values of gene ages (center line) was assessed using an FDR-corrected permutation test. (D) Mosaic plot showing that the species-level genes (rank 9) are responsible for the enrichment of young genes in the sex chromosome.*

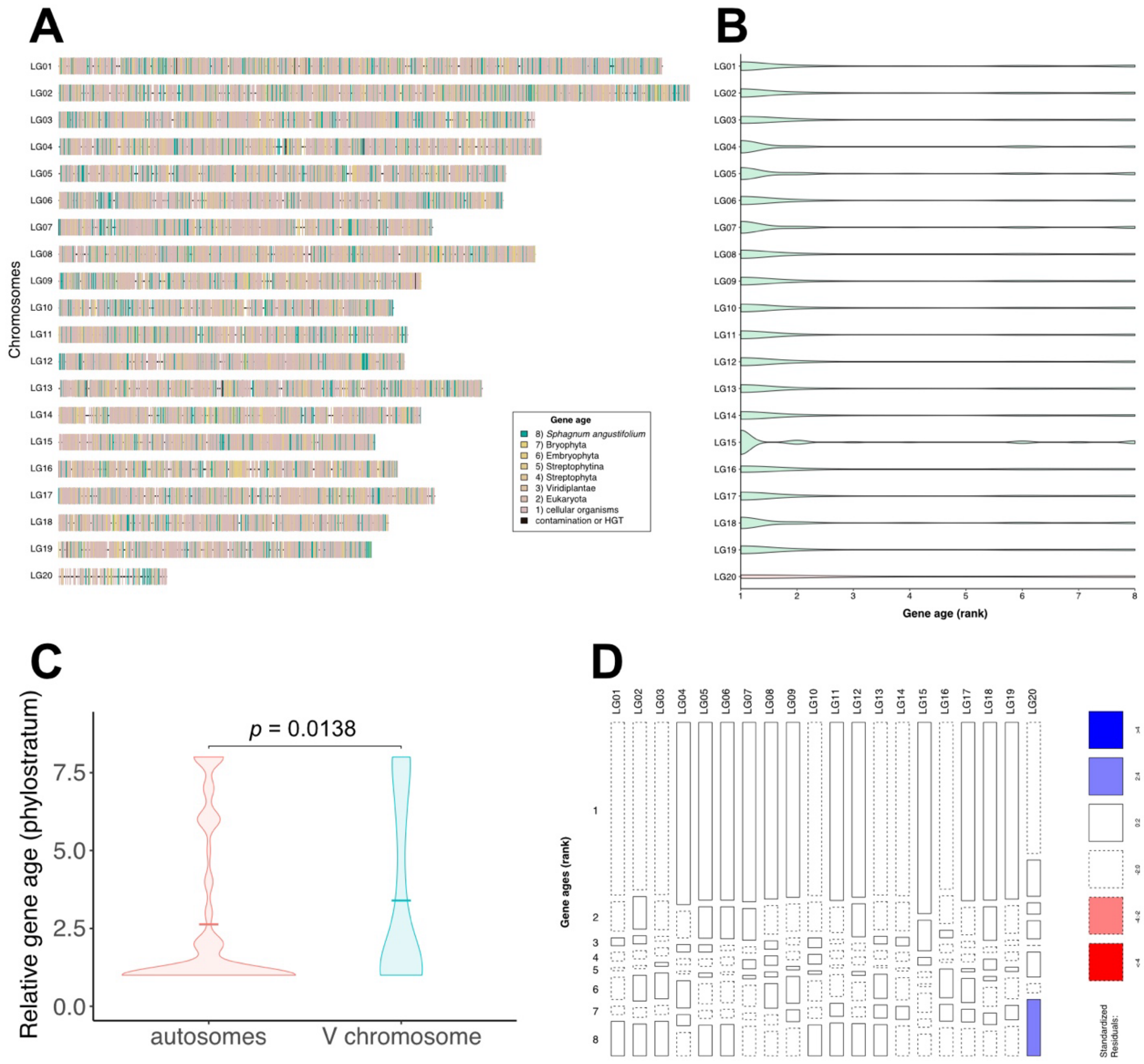

Supplementary Figure 13. *Gene ages across the Sphagnum angustifolium genome. (A) Distribution of relative gene ages across the chromosomes of Sphagnum angustifolium. (B) The V sex chromosome (LG20; red) has a significantly higher proportion of young genes and a lower proportion of old genes when compared to the autosomes (green; see Supplementary Table 14). (C) Violin plot showing the relative gene age ranks (higher ranks equate to younger ages) of the TRGs between the autosomes and the V chromosome. Statistically significant difference in mean values of gene ages (central line) was assessed using an FDR-corrected permutation test. (D) Mosaic plot showing that the species-level genes (rank 8) are responsible for the enrichment of young genes in the sex chromosome.*

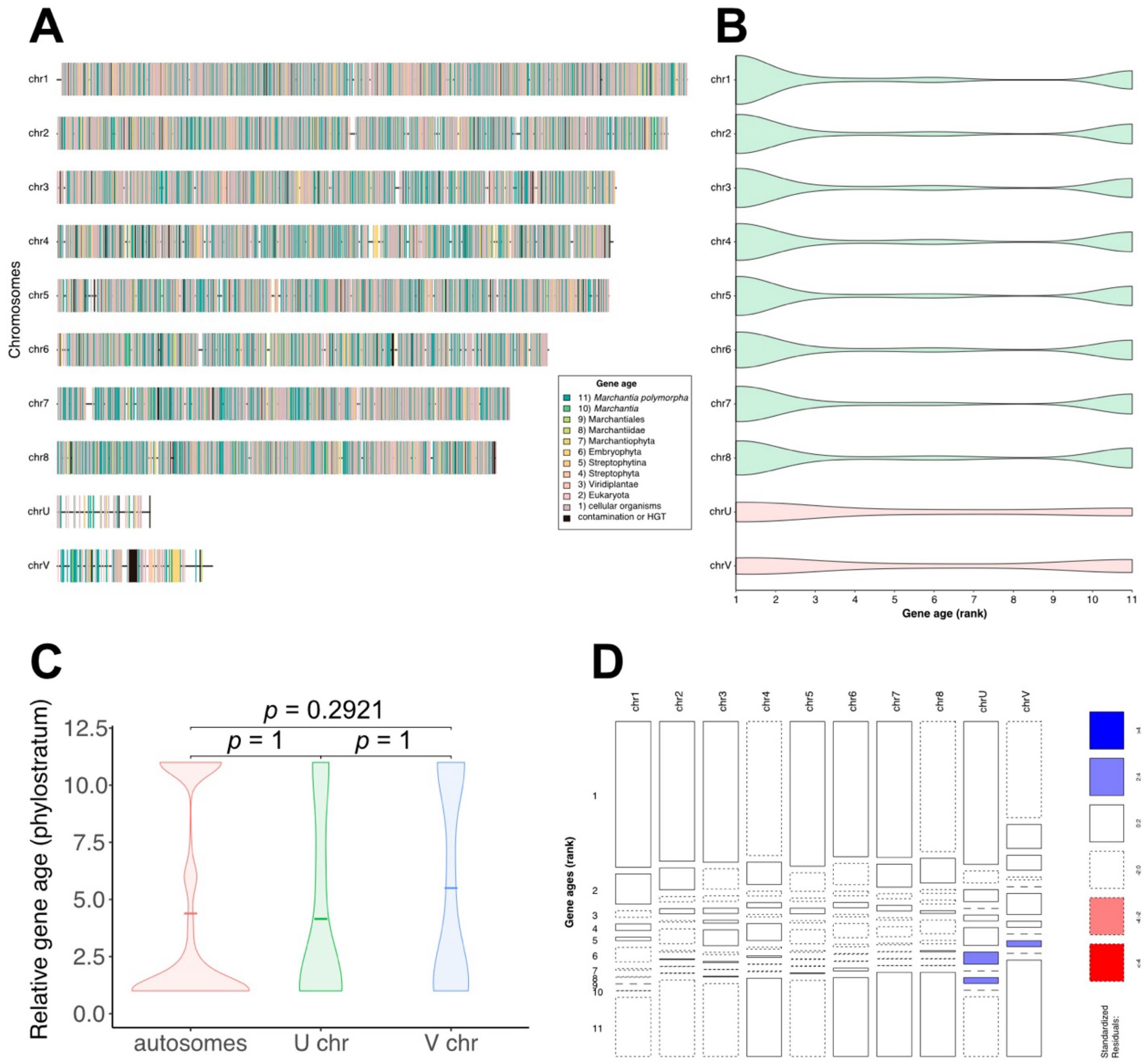

Supplementary Figure 14. *Gene ages across the Marchantia polymorpha genome. (A) Distribution of relative gene ages across the chromosomes of Marchantia polymorpha. (B) The U/V sex chromosomes (chrU and chrV; red) show non-significant differences in gene age distribution when compared to the rest of the chromosomes (green; see Supplementary Table 14). (C) Violin plot showing the relative gene age ranks (higher ranks equate to younger ages) of the TRGs between the autosomes and the sex chromosomes. No statistically significant differences were found in median values of gene ages (FDR-corrected permutation tests). (D) Mosaic plot showing non-significant differences between the sex chromosomes and the autosomes.*

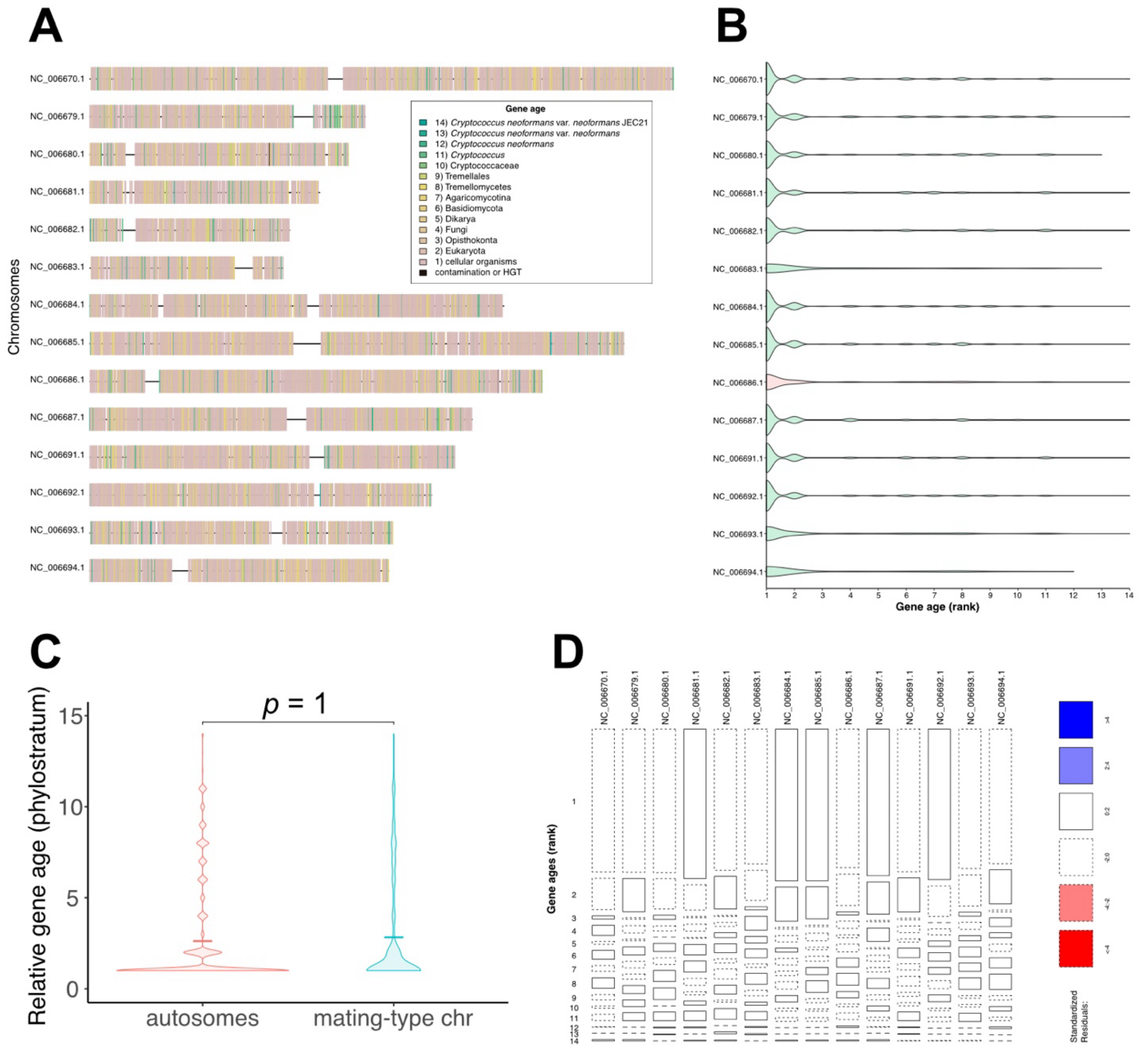

Supplementary Figure 15. *Gene ages across the Cryptoccocus neoformans var. neoformans JEC21 genome. (A) Distribution of relative gene ages across the chromosomes of Cryptoccocus neoformans. (B) The mating-type chromosome (NC_006686.1; red) shows non-significant differences in gene age distribution when compared to the rest of the chromosomes (green; see Supplementary Table 14). (C) Violin plot showing the relative gene age ranks (higher ranks equate to younger ages) of the TRGs between the autosomes and the mating-type chromosome. No statistically significant difference was found in the mean values of gene ages (FDR-corrected permutation tests). (D) Mosaic plot showing no discernible pattern of gene age distribution in any of the chromosomes.*

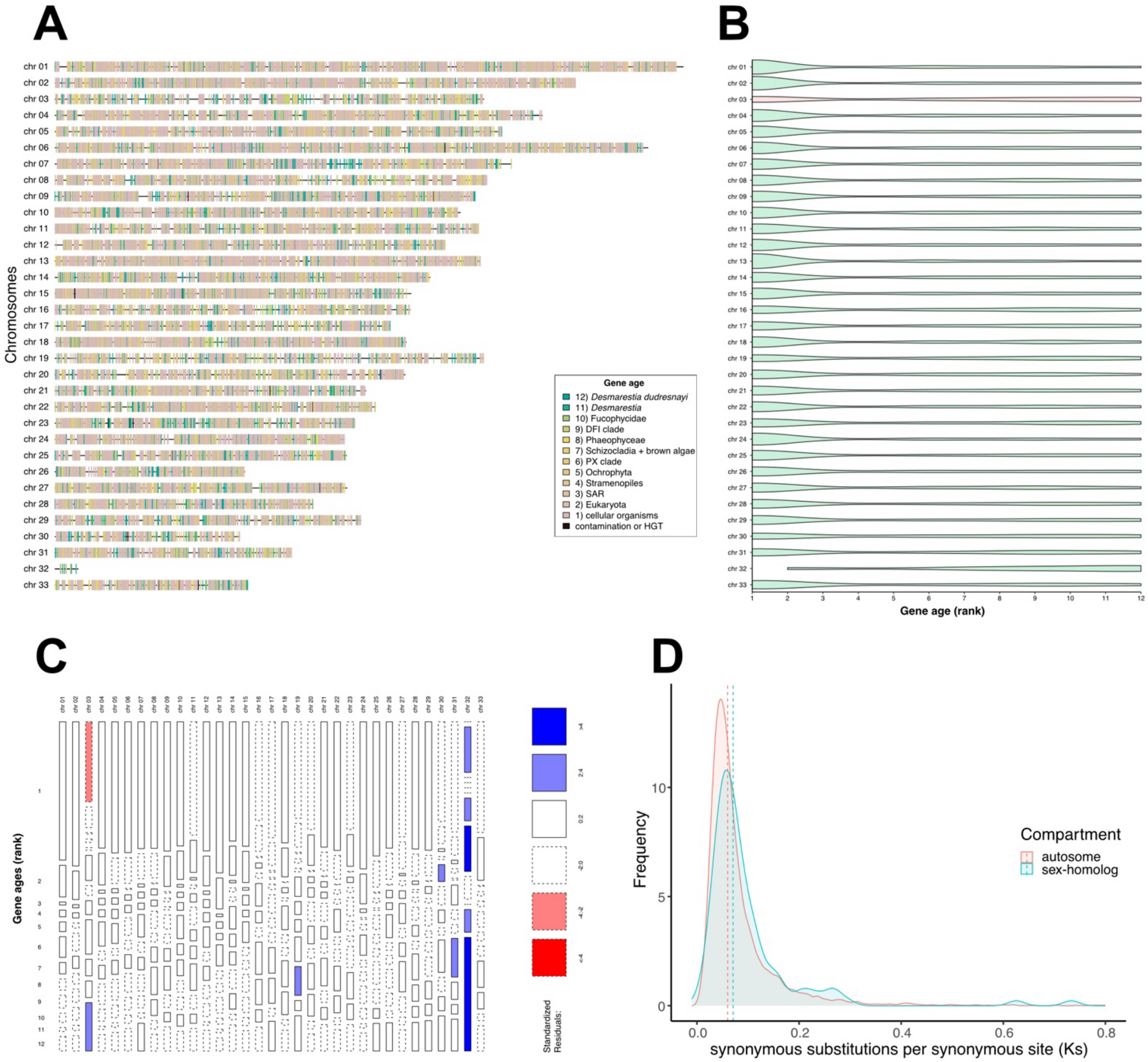

Supplementary Figure 16*. Gene ages across the D. dudresnayi genome. (A) Distribution of relative gene ages across the chromosomes of Desmarestia dudresnayi. (B) The U/V-homolog in D. dudresnayi (chr 03; red) has a significantly higher proportion of young genes and a lower proportion of old genes when compared to most of the other chromosomes (green; see Supplementary Table 14). (C) Mosaic plot showing that the species-level genes (rank 12) are responsible for the enrichment of young genes in the U/V-homolog. (D) The inter-species Ks values are similar in the U/V-homolog when compared to the other chromosomes (see Supplementary Table 17).*

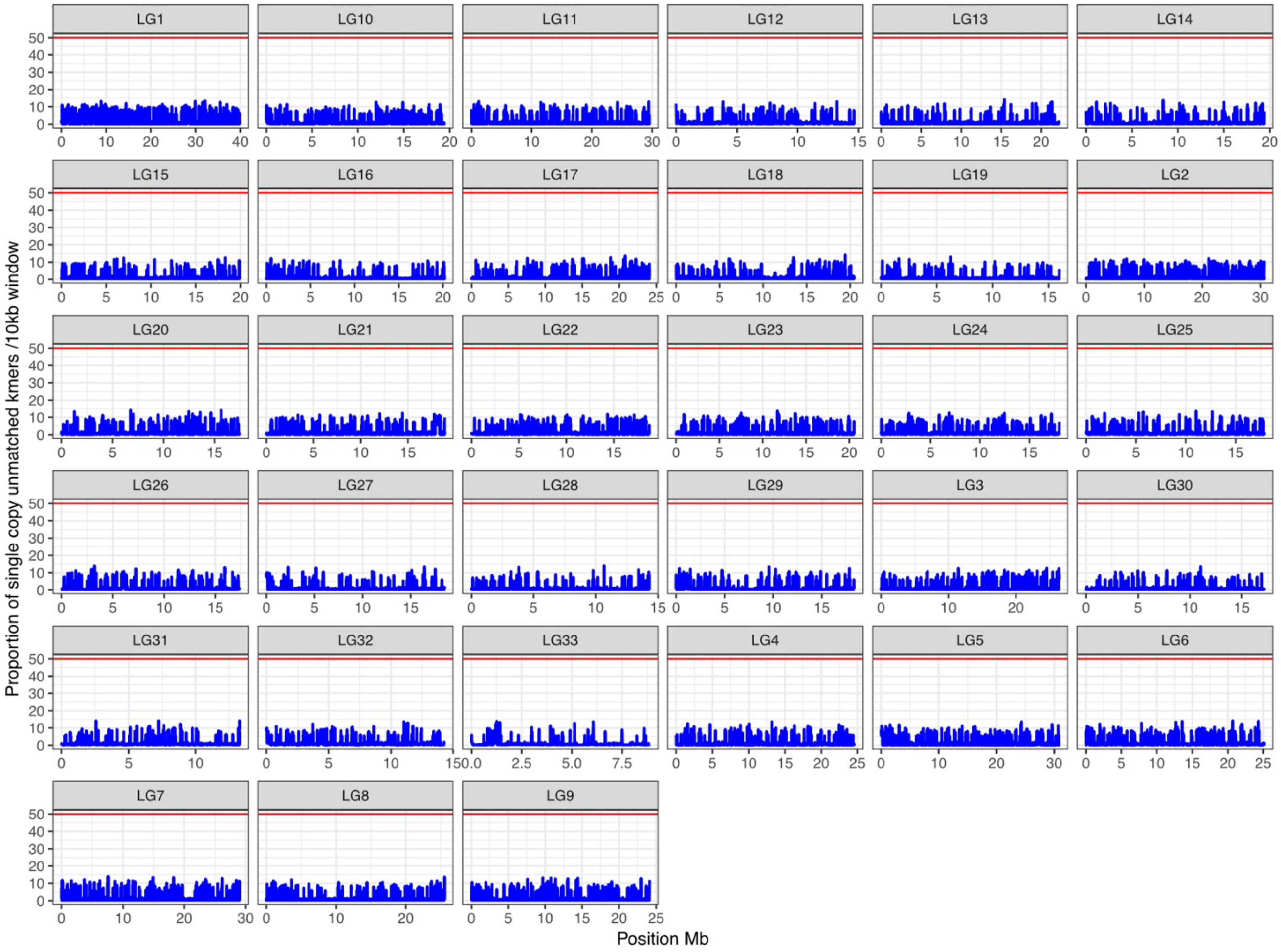

Supplementary Figure 17. *No detectable sex-determining region in the male genome of Fucus serratus. None of the chromosome-level scaffolds display differences in k-mer coverage between the male and the female genomes of F. serratus.*

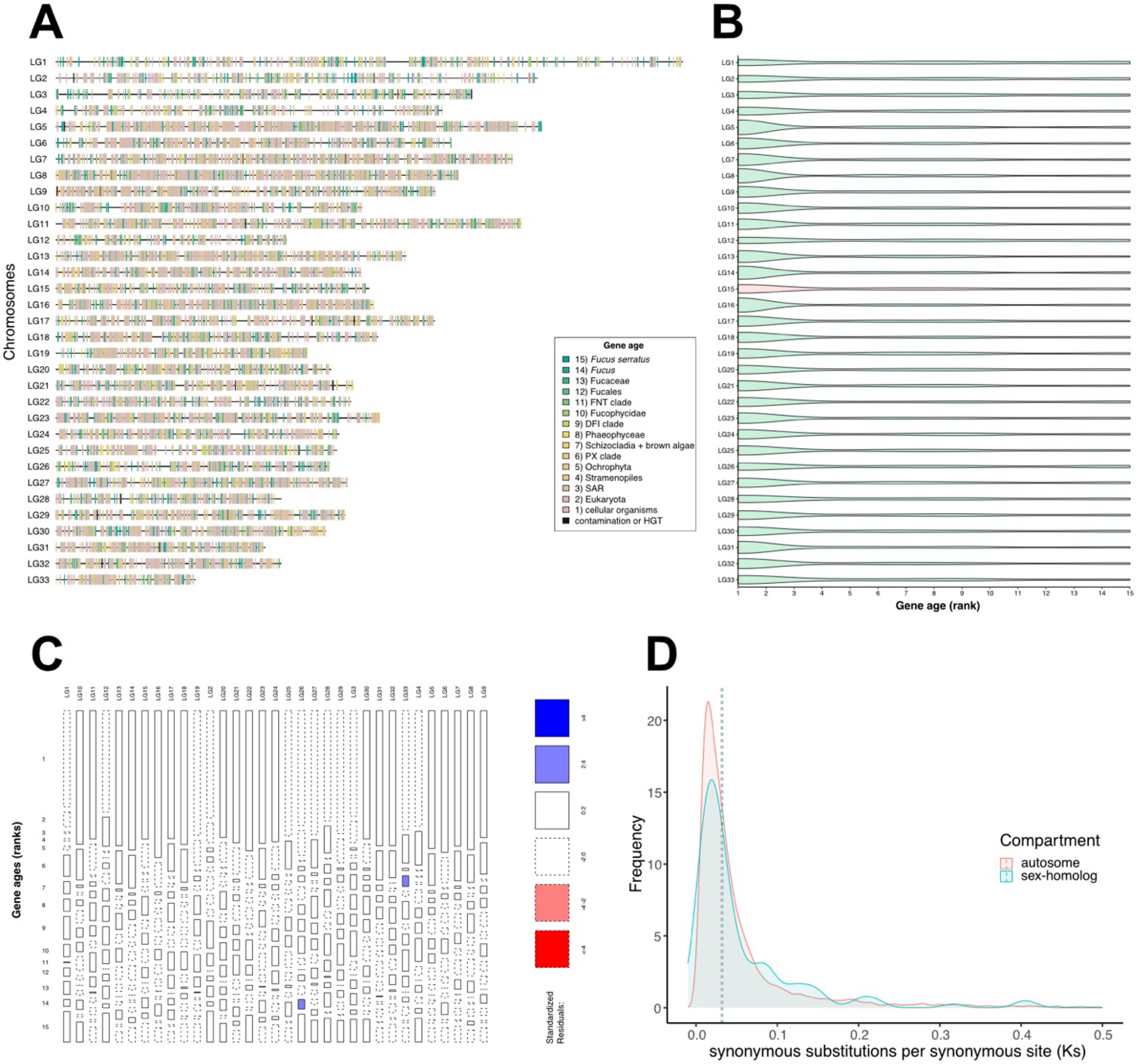

Supplementary Figure 18. *Gene ages across the F. serratus genome. (A) Distribution of relative gene ages across the chromosomes of Fucus serratus. (B) The U/V-homolog in F. serratus (LG15; red) shows no significant differences in gene age distribution when compared to the rest of the chromosomes (green; see Supplementary Table 14). (C) Mosaic plot showing no discernible pattern of gene age distribution in any of the chromosomes. (D) The inter-species Ks values are similar in the U/V-homolog when compared to the other chromosomes (see Supplementary Table 17).*
